# Challenges in Anesthesia in Wilson’s Disease: A Systematic Review of the Existing Literature

**DOI:** 10.7759/cureus.33334

**Published:** 2023-01-04

**Authors:** Ajisha Aravindan, Kandukuri Shiva Priya, Sumit Roy Chowdhury, Priyankar K Datta

**Affiliations:** 1 Anaesthesia and Critical Care, All India Institute of Medical Sciences, New Delhi, New Delhi, IND; 2 Neuroanaesthesiology and Critical Care, All India Institute of Medical Sciences, New Delhi, New Delhi, IND

**Keywords:** regional anesthesia, general anesthesia, anesthesia, spinal anesthesia, wilson’s disease

## Abstract

Wilson’s disease (WD) is a disorder of copper metabolism presenting with a wide variety of organ dysfunctions affecting predominantly the neurological, hepatic, and hematological systems. Due to its multi-systemic nature, administering anesthesia to a patient with WD is challenging and requires an in-depth knowledge of the disease pathophysiology. A systematic search for literature pertaining to the anesthetic management of patients with WD yielded 19 case reports, which we have included in this review to explore and summarize peri-operative concerns and the safe anesthesia practices in this condition.

## Introduction and background

Wilson’s disease (WD) is a rare genetic disorder of copper metabolism with a prevalence of around 0.5 in 100,000. It is an autosomal recessive disorder, with the underlying pathology being mutation of the gene encoding ATP7B protein at chromosome 13q14. This protein is involved in the incorporation of copper into its transport protein - ceruloplasmin. In the absence of this protein, excessive amounts of free copper are released into the plasma and are deposited in tissues of the liver, kidney, and brain, causing oxidative damage through a chemical process called Fenton reaction. Neuropsychiatric manifestations are the commonest clinical presentation in WD [1]. However, the complex pathophysiology of WD means that patients may present with a varied spectra of clinical symptoms including hepatic, renal, ocular, and hematological dysfunction. Rarely, WD can be sporadic in origin as well [2].

Hepatic dysfunction in WD may range from chronic active hepatitis to fulminant hepatic failure, with most patients presenting with chronic liver disease and cirrhosis. The most common neuropsychiatric manifestation of WD is asymmetric tremors, but it may also present with ataxia, mask-like facies, excess salivation, personality changes, emotional liability, and impulsiveness. The chronic neurodegenerative process may lead to dystonia in the form of arthropathies, which are of importance during positioning for surgery [3,4]. When left untreated, WD is fatal. However, with the initiation of appropriate copper chelation, absorption blocker, and other anti-copper therapies early, the life expectancy can match that of the general population [5].

The multi-systemic nature of WD poses a significant challenge for the anesthetist, requiring meticulous peri-operative planning and management. The degree of liver dysfunction plays a vital role in the planning of the anesthetic technique as most of the anesthetic drugs are metabolized by the liver. Due to the relatively rare nature of the disease, there is a dearth of knowledge regarding anesthetic concerns and safe practices in this condition. There are a few case reports describing anesthetic management of patients with WD. There is no available literature that summates the existing evidence to guide the peri-operative management in WD. In this article, we have reviewed published articles on the management of patients with WD for various non-liver transplant surgeries. Although reports of patients with WD for liver transplant (LT) surgeries have not been included in this review, we have emphasized the points important during LT which are exclusive for patients with WD.

## Review

Search strategy

**Figure 1:**
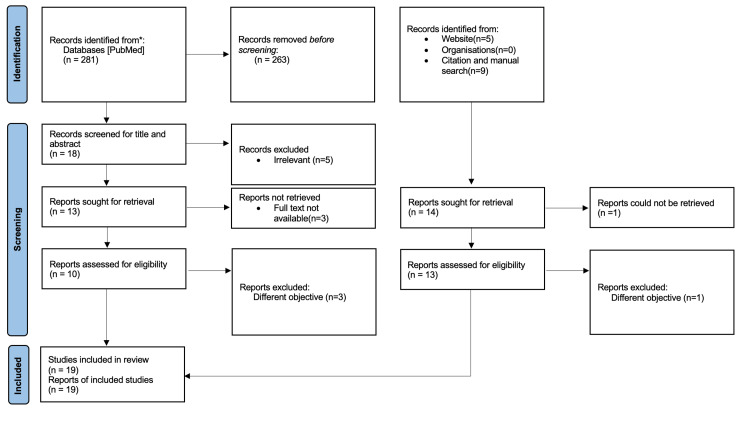
PRISMA flow diagram showing the process of selection of studies

PubMed and Google Scholar databases were searched using the following keyword algorithm: ((Wilson disease) OR (Wilsons disease) OR (Wilson's disease)) AND ((Anesthesia) OR (Anaesthesia)). Peer-reviewed articles of which full-text versions were available in English language were selected. In case full-text links were unavailable, authors were not contacted to obtain them. In addition, a manual search of the citations of the selected articles was performed to look for any articles that may have been missed. Reports on the anesthetic management of LT in patients with WD were excluded. Finally, 19 articles describing anesthetic management of 19 different cases of WD were included in this review. PRISMA guidelines were followed while preparing this review [6]. The search strategy is depicted in the flow chart in Figure 1.

Review of the published cases

The most common s for the requirement of anesthesia were obstetric and gynecological procedures (n = 6/19) including cesarean section (n = 4/19) and termination of pregnancy. Other described surgical procedures included scoliosis correction, orthopedic procedures for trauma, and neurosurgical procedures for traumatic brain injury. The age range of the patients varied from 4 years to 53 years. Most of the patients already came with the diagnosis of WD, and the disease duration varied between 9 months and 25 years. One patient was diagnosed on the current hospital admission. The patients were on some form of treatment for WD, which included D-penicillamine, zinc, trientine, and low-copper diet. Few patients had stopped D-penicillamine during pregnancy. Only one patient had already undergone an LT. Deranged liver function in the form of elevated liver enzymes and serum bilirubin, and radiological evidence of liver parenchymal disease were the commonest abnormalities detected. Clinical features of chronic liver disease including ascites, generalized edema, jaundice, and bleeding diatheses were found in 10 patients. Portal hypertension and hepato-splenomegaly were frequently detected. Neuropsychiatric symptoms were reported in seven patients. Hematological abnormalities including anemia and thrombocytopenia were present in six patients. All kinds of anesthetic management ranging from general anesthesia (GA), spinal anesthesia (SA), regional anesthesia (RA), to monitored anesthesia care (MAC) have been described in the literature. Overall, 11 patients received GA, three patients received nerve blocks, and three patients received SA. Combined spinal epidural anesthesia and conscious sedation were administered in one patient each. Propofol was the induction agent used in all the cases that received GA. Ketamine was used along with propofol in only one patient. Fentanyl was the most commonly used opioid (n = 7/11 cases). Remifentanil was used in two cases. One patient received pethidine. In most cases of GA (n = 6/11), atracurium was used as a muscle relaxant, while rocuronium and vecuronium were used in 1 case each. Succinylcholine was also used in one patient. Two patients did not receive any muscle relaxant. Isoflurane was the most commonly used volatile agent for GA (n = 5/11). There is one reported case of total intravenous anesthesia (TIVA). For SAB, bupivacaine was the sole local anesthetic drug used for all cases. There were no significant intra-operative or postoperative complications reported in any of the cases (Table 1).

**Table 1:** Review of the case reports describing anesthetic management in patients with Wilson's disease POG, period of gestation; FGR, fetal growth restriction; DIC, disseminated intravascular coagulation; AT-III, anti-thrombin 3; LSCS, lower segment Caesarean section; GA, general anesthesia; ICU, intensive care unit; USG, ultrasonogram; IV, intravenous; PNS, peripheral nerve stimulator; PACU, post-anesthesia care unit; WD, Wilson's disease; CT, computed tomography; K-F, Kayser-Fleischer; PT, prothrombin time; CLD, chronic liver disease; EHPVO, extra hepatic portal venous obstruction; TAP, transversus abdominis plane; SA, spinal anesthesia; CSE, combined spinal epidural; TIVA, total intravenous anesthesia; IM, intramuscular; AST, aspartate aminotrasferase; LFT, liver function test

Study	Age (years)/sex	Duration of disease	Clinical setting and pre-operative status of Wilson’s disease	Treatments	Remarkable investigations	Type of surgery	Anesthetic technique used	Intra-operative concerns	Post-operative outcome
Saito et al. [7]	24/F	21 years	Parturient. Patient had undergone liver transplant at 20 years of age. Hospitalized at 25 weeks’ POG with thrombocytopenia and FGR. Developed rapid onset ascites and edema subsequently. Diagnosed as DIC with oliguria, thrombocytopenia, respiratory distress, liver dysfunction, and severe coagulopathy.	Anti-copper therapy continued in pregnancy. AT-III concentrates, platelets, cryoprecipitate, and fibrinogen concentrates in hospital	ALT, AST normal. Low fibrinogen, AT-III levels	Emergency LSCS	GA. Induction drugs: propofol, remifentanil, rocuronium. N_2_O, sevoflurane used for maintenance of anesthesia until delivery. After delivery, continuous propofol and remifentanil infusion used.	Blood loss requiring transfusion of blood products	Pulmonary edema requiring mechanical ventilation in ICU. Discharged after 15 days. Coagulation status normalized 40 days after delivery.
Wan et al. [8]	26/F	15 years	Parturient. Bilateral pitting edema of lower limbs.	Penicillamine stopped 3 months before pregnancy and started on zinc.	Total bilirubin: 27.3 μmol/L; platelet: 56×10^9^/L. USG of the abdomen: coarse liver echo-texture, splenomegaly	Elective LSCS	GA: Propofol, remifentanil, succcinyl choline, sevoflurane	Unremarkable	Uneventful
Tokgöz et al. [9]	24/M	18 years	Fracture humerus. Difficulty in walking and speech, had tremors and involuntary muscle contractions.	Penicillamine and low-copper diet for last 3 years	Nil	Emergency surgical fixation	1mg IV midazolam followed by PNS-guided infra-clavicular nerve block - bupivacaine.	Unremarkable	Surgery uneventful. Discharged from PACU after 1 hour.
De Souza Hobaika [10]	53/F	25 years	Rotator cuff injury. Dysarthria, tremors of upper extremities.	Penicillamine	Nil	Arthroscopic rotator cuff repair	PNS-guided inter-scalene block - ropivacaine. Midazolam for sedation.	Unremarkable	Unremarkable
Baykal et al. [11]	4/M	18 months	Traumatic depressed skull fracture. No symptoms of WD.	Zinc, penicillamine	Unremarkable except chest X-ray and CT of the thorax showing opacity and nodular infiltration in the right lower lobe of the lungs, respectively.	Emergency neurosurgery	GA: Propofol, fentanyl, atracurium, Isoflurane	Unremarkable	No post-operative worsening of liver or renal functions. Chest X-ray was normal on day 3. Discharged on day 7. Clinical examination and laboratory tests on follow-up at 1 month were normal.
Bhaskar Rao et al. [12]	29/F	10 years	Pregnant. K-F rings, bilateral pedal edema.	Zinc, penicillamine	Mild elevated liver enzymes, low platelets, and elevated PT. Hepatosplenomegaly, dilated portal vein.	Therapeutic abortion	Conscious sedation: midazolam, glycopyrrolate, propofol infusion, fentanyl before cervical dilation. Tramadol for post-operative pain relief.	Uneventful	Uneventful
Amrinder Kaur et al. [4]	40/F	8 years	Infected orthopedic implant in arm. Depression, slurred speech, fine tremors, K-F ring.	Zinc, trihexyphenidyl, propranolol, glycopyrrolate	Within normal limits	Implant removal	USG-guided inter-scalene block - levobupivacaine and dexamethasone	Uneventful	Uneventful
Mohanty et Al. [13]	21/F	3 years	Parturient. CLD with history of EHPVO, hypersplenism, recurrent jaundice, platelet transfusions.	Zinc	Thrombocytopenia, mild ascites, liver parenchymal disease, and massive splenomegaly with dilated portal vein	Elective LSCS	GA with B/L TAP block. Propofol, atracurium, N_2_O, isoflurane. 4 units of platelets during induction. Bupivacaine in TAP block.	Uneventful	Uneventful. Discharged on day 4
Athar et al. [14]	23/M		Contracture of knees.	Penicillamine		Contracture release	SA: bupivacaine heavy	Uneventful	Uneventful. Patient discharged after a week.
Kitano [15]	34/M	18 years	Limb deformity. Speech disorder, learning difficulty. Previous history of surgery in which delayed awakening (half a day) after GA was reported by the patient.		Severe liver dysfunction	Heel and foot arthroplasty	CSE with monitored anesthesia care: bupivacaine in SAB, intra-operative dexmedetomidine infusion, post-operative continuous epidural infusion.	Uneventful	Uneventful
Agrawal et al. [16]	12/F	6 years	Gall stone disease. No other symptoms of WD.	Penicillamine, zinc	Nothing remarkably abnormal	Laparoscopic cholecystectomy	GA: propofol, fentanyl, atracurium, isoflurane. Paracetamol	Surgery uneventful	No post-operative deterioration of liver or renal function.
Gupta et al. [17]	22/M	2 years	Scoliosis. No other symptoms of WD.	Penicillamine, zinc	Within normal limits	Scoliosis correction	GA. Induction: fentanyl, propofol, atracurium; fentanyl and propofol infusion in TIVA. Tranexamic acid, ondansetron, dexamethasone, ketorolac.	1.5L blood loss, 4 units each of PRBC, FFP, and platelet concentrates transfused. No hemodynamic instability, extubated on table.	Post-operative investigations normal. No deterioration of any clinical or laboratory parameters.
Bhokare et al. [18]	13/	7 years	Difficulty in maintaining airway and clearing secretions. Dystonia, dysarthria, muscle wasting, bronchospasm, and oro-mandibular muscle stiffness. Bilateral basal crepitations, wheeze, and chest wall rigidity.	Was on penicillamine but non-compliant for 2 years.	Elevated liver enzymes, low protein levels, low serum calcium	Emergency tracheostomy	GA. Premedication with injection glycopyrrolate and midazolam; dexamethasone and hydrocortisone given prophylactically; induced with propofol; fentanyl avoided as patient had pre-existing chest rigidity; muscle relaxants also avoided; maintained with O_2_, N_2_O, and propofol boluses; salbutamol puffs given.	Uneventful	Uneventful
Chakravarthy et al. [19]	19/F	3 years	Parturient. No symptoms of WD.	Not on any medications since 5th month of pregnancy. Previously on combination of levodopa-carbidopa, trientine, and zinc	Within normal limits	Emergency LSCS	SA: bupivacaine and fentanyl. Post-operative analgesia: fentanyl, diclofenac, paracetamol	Uneventful	Uneventful
Bhalerao et al. [20]	28/F	13 years	Splenomegaly with hypersplenism. Fever with chills and dizziness for 3 months, pallor. Ophthalmic, neuropsychiatric status normal.	Penicillamine, zinc	Anemia, thrombocytopenia. USG of the abdomen: liver parenchymal disease and splenomegaly	Splenectomy	GA. Premedicated with glycopyrrolate 0.2 mg IM half an hour before the procedure, followed by ondansetron 4 mg, midazolam 1 mg, and fentanyl 50μg intravenously. Induction: Propofol, Atracurium. Maintenance: N_2_O, isoflurane.	1.5L blood loss, required transfusion of blood products hemodynamically stable.	Uneventful
Nanjangud and Prasad [21]	15/F	Diagnosed with Wilson's disease on admission	Admitted to psychiatric ward with progressive slurring of speech, gait disturbance, insomnia, altered behavior, tremors, abnormal movements, hypertonia of limbs, focal seizures, fluctuating consciousness, inattention, inappropriate laughter and cry. Later found to have shoulder dislocation and abscess.	Initially on risperidone and carbamazepine. Steroids, penicillamine, trihexyphenidyl, quetiapine, levetiracetam initiated on suspicion of WD.	Mildly elevated liver enzymes, increased serum and urinary copper levels	Reduction of shoulder dislocation and drainage of shoulder abscess	GA: fentanyl, propofol, atracurium, isoflurane, N_2_O; plus PNS-guided inter-scalene block - bupivacaine; paracetamol.	Uneventful	No deterioration of liver function or neurological status. Gradually extra-pyramidal symptoms decreased, speech improved on treatment.
Kurdi et al. [3]	33/F	9 months	Missed abortion. Pallor, pedal edema, icterus, facial puffiness, abdominal distension.	Propranolol, zinc, frusemide - stopped for 5 months	Anemia, high total bilirubin, elevated AST; USG: mild hepatosplenomegaly, dilated portal vein, ascites.	Elective suction evacuation of retained products of conception	GA. Propranolol 20 mg in the morning. Premedication: glycopyrrolate; sedation with fentanyl, ketamine, propofol; maintained on spontaneous ventilation with O_2_ and N_2_O.	Uneventful	Uneventful.
Maharjan [22]	14/F	2 years	Femur fracture. Difficulty in standing, walking, speaking, abnormal posturing, tremors, rigidity with flexion of the limbs, irritable, uncooperative, unable to speak	Initially penicillamine, later trientine, zinc. trihexyphenidyl, clonazepam.	Slightly deranged LFTs	Femur fracture fixation	GA: Propofol, pethidine, vecuronium, isoflurane	Uneventful	Uneventful
Inanoglu et. al. [23]	18/M	5 years	Femur fracture. Progressive deterioration of consciousness, speech and movement, lack of motor co-ordination, spasticity and dystonia of all four limbs, drooling, plastic dystonic rigidity of knees.	Trientine, pyroxidine, thiamine, baclofen, zinc	Nothing remarkable except elevated AST at 110 IU/L	Intra-medullary nailing	SA: 3ml hyperbaric bupivacaine	Uneventful	Uneventful. Discharged on post-operative day 4

Systemic manifestations of Wilson’s disease relevant to anesthesia

In the brain, copper is found predominantly in the basal ganglia and the hippocampus. Excess copper deposition in these regions in WD leads to clinical manifestations similar to Parkinsonism, including tremors, ataxia, akinesia, rigidity, and dystonia, often leading to contractures. The patients can also have bulbar symptoms, drooling of saliva, spasticity, micrographia, dyslalia, hypomimia, speech disorders, and a variety of movement disorders such as chorea, myoclonus, and athetosis. Poor scholastic performance, depression, paranoia, schizophrenia, and behavioral changes are the common psychiatric symptoms. It has been observed that visual and auditory evoked potentials are altered in WD patients, suggesting damage of the brain stem, sensory tracts, and cortex. However, peripheral nerve conduction and the somatic-sensory evoked potentials are usually normal. Autonomic dysfunction may be present [1,4,24].

Hepatic impairment can be of a wide spectrum, ranging from mild histological or biochemical changes to acute liver failure, fulminant hepatitis, chronic active hepatitis, and cirrhosis. LT may be deemed appropriate in patients with severe hepatic impairment [25,26]. Patients who have primary liver dysfunction usually become symptomatic at the end of the first decade of life, while those who have primary neuropsychiatric involvement present later. The second group of patients will have some degree of hepatic derangement during presentation. In general, patients with primary neuropsychiatric symptoms usually have poorer quality of life and shorter survival than those who have undergone LT for primary hepatic involvement [5].

Kayser-Fleischer (K-F) rings and sunflower cataracts are two classical ophthalmologic findings [25].

In the cardiovascular system, concentric left ventricular remodelling leads to diastolic dysfunction. In vitro studies have found copper chloride to reduce cardiac rate, action potential duration, and amplitude [2]. Patients with WD are at a higher risk of developing cardiomyopathy, conduction defects, congestive heart failure, atrial fibrillation, and ventricular arrhythmias [27]. The presence of autonomic dysfunction may further worsen hemodynamic reserve.

WD may rarely present as respiratory failure from muscle rigidity and weakness. The pulmonary involvement resulting from liver and cardiac dysfunction may further contribute to respiratory insufficiency and failure [2].

Hematological manifestations include defective hematopoiesis leading to microcytic hypochromic anemia, and hemolysis due to reduced erythrocyte membrane deformability as a result of oxidative stress, leading to Coomb’s negative hemolytic anemia and indirect hyperbilirubinemia [2,25].

Renal dysfunction seen in WD can be due to the direct toxic effect of free copper as well as due to the indirect effect of hemoglobinuria due to intravascular hemolysis [2,24,25]. WD may be associated with endocrine failure and metabolic bone disease [28].

Preoperative assessment and management

General physical examination of the patient with emphasis on relevant systemic involvement is the goal of preoperative assessment. Liver and kidney functions need careful assessment as most of the anesthetic drugs are excreted through these systems. As patients with WD often have significant cardiac involvement, any cardiovascular symptoms or abnormalities on electrocardiogram warrant further investigation. History of tremors, ataxia, and other neuropsychiatric issues need to be well documented. Patients with dysarthria and bulbar involvement are at a high risk of aspiration. Apart from routine fasting orders, gastric acid suppressants, prokinetic agents, and other anti-aspiration measures are important considerations in them. Whether to continue the medications for WD in the peri-operative period is not clearly specified in the literature. In the absence of a clear guideline, shared decision-making between the neurologist, the anesthetist, and the surgeon may be a safe and wise approach.

In the presence of severe liver derangement, elective non-essential surgeries should be deferred. WD with acute liver failure may pose additional challenges in the form of rapidly worsening sensorium, cerebral edema, renal injury, and coagulopathy, which is unresponsive to vitamin K therapy. These patients will be critically ill and will require an intensive care therapy with a dedicated multidisciplinary management. Renal replacement therapy is frequently required. In the presence of acute liver failure, close attention is needed in managing cerebral edema and raised intracranial pressure (ICP). Non-invasive monitoring of cerebral perfusion monitoring using near-infrared spectroscopy or trans-cranial Doppler can be employed when severe coagulopathy precludes invasive ICP monitoring. The management of patients with chronic liver disease with WD is not significantly different from what is followed in other patients with chronic liver disease, with careful attention to other systemic involvement [2,5].

Patients with WD are prone to developing dystonic storm characterized by sudden onset of hyperthermia, hemodynamic instability, sweating, dysphagia, dysarthria, and even respiratory failure. D-penicillamine and zinc may precipitate acute dystonia as well [29].

It is prudent to counsel related family members of the patient to undergo screening for WD, if not already undertaken.

Intraoperative management

Musculoskeletal contractures as a result of neurodegenerative disease may make airway management and patient positioning difficult. GA [7,8], neuraxial anesthesia [14,15], and regional block techniques [4,10] have been successfully used in patients with WD.

In case GA is planned, care has to be taken in terms of both choice of the drug used and the dosage, as both inhalational and intravenous agents can have accentuated effects and prolonged action due to reduced protein binding and slow metabolism. Propofol may be preferred for the induction of anesthesia as it is not significantly protein bound and is unlikely to produce an exaggerated response. It may be judicious to avoid ketamine and etomidate in patients with WD due to their potential to precipitate seizures [2,4,8,30].

Duration of action of non-depolarizing muscle relaxants may be increased due to higher free fraction, lower metabolism, and increased sensitivity. Cisatracurium can be considered as a better option for skeletal muscle relaxation because of its organ-independent metabolism and lower potential for production of the epileptogenic metabolite laudanosine. Although there is one report of safe use of succinylcholine while administering GA for a cesarean section, it is better avoided in patients with polyneuropathy and myopathy due to the potential for precipitating hyperkalemia. In the presence of significant liver or renal dysfunction, it is judicious to avoid rocuronium or vecuronium as there may be undue prolongation of paralysis [2,3,17]. In patients with liver failure for emergency LT, ICP management is crucial [5].

Based on the current evidence, the preference for RA techniques over GA, or vice-versa, remains unclear and should be decided on a case-to-case basis. Since lower motor function is usually unaffected, RA can be safely employed as the sole anesthetic technique or as an adjuvant to GA. However, any pre-existing neuromuscular weakness should be documented prior to RA. Although there are multiple reports emphasizing the safety of RA and neuraxial anesthesia in patients with pre-existing neurological dysfunction, a case-specific careful approach involving a senior anesthesiologist and a neurologist is logical. Autonomic instability may result in exaggerated hemodynamic swings following neuraxial anesthesia. Coagulopathy due to liver dysfunction should always be kept in mind while using regional techniques. Low protein binding may increase local anesthetic toxicity in patients with chronic liver disease [4,9,11,14].

Postoperative care

Dystonia is a common feature of WD and may be exaggerated in the postoperative period, resulting in airway obstruction and respiratory insufficiency. Respiratory depression caused by prolonged action of anesthetic agents and pre-existing respiratory muscle weakness may precipitate post-operative respiration failure. Metoclopramide should be avoided for prophylaxis and treatment of post-operative nausea and vomiting due to the potential for worsening extra-pyramidal symptoms. The presence of hematological dysfunction means that patients with WD may be immunosuppressed, and postoperative sepsis is a grave potential complication in these patients [2-4,17]. Acute worsening of neuropsychiatric symptoms after GA has been reported [31]. Patients with pre-existing cognitive impairment may be exceptionally prone to postoperative delirium and agitation [3,4]. To prevent worsening of symptoms, copper levels need to be maintained in the safe range by continuous pharmacotherapy, which highlights the importance of restarting the medications the patient was receiving as early as possible after surgery [11].

Intensive care management

In patients with WD crisis and fulminant hepatic failure, apart from routine ICU management, lowering serum copper levels becomes crucial. Therapeutic plasma exchange and albumin dialysis are effective modalities for copper removal [32].

## Conclusions

WD is a challenge for the anesthesiologist. Appropriate management requires detailed knowledge of the organ systems involved and needs to be tailored according to the individual patient's clinical profile. However, owing to the relative rarity of the condition, most of the information regarding the anesthetic management of WD is from isolated case reports. The paucity of scientific data in this regard is concerning. We therefore believe that an international registry of this rare condition is needed, which will facilitate the gathering of useful information which can help both patients and peri-operative clinicians involved in their care.
